# Antioxidant Activity and Inhibitory Potential of* Cistus salviifolius* (L.) and* Cistus monspeliensis* (L.) Aerial Parts Extracts against Key Enzymes Linked to Hyperglycemia

**DOI:** 10.1155/2017/2789482

**Published:** 2017-01-01

**Authors:** Karima Sayah, Ilias Marmouzi, Hanae Naceiri Mrabti, Yahia Cherrah, My El Abbes Faouzi

**Affiliations:** Faculté de Médecine et de Pharmacie, Laboratoire de Pharmacologie et Toxicologie, l'Équipe de Pharmacocinétique, Mohammed V University in Rabat, BP 6203, Rabat Instituts, Rabat, Morocco

## Abstract

*Cistus* genus (Cistaceae) comprises several medicinal plants used in traditional medicines to treat several pathological conditions including hyperglycemia. These include* Cistus salviifolius *L. (CS) and* Cistus monspeliensis *L. (CM), still not fully explored as a source of metabolites with therapeutic potential for human diseases. In this study, the antioxidant *α*-amylase and *α*-glucosidase enzyme inhibitory effects of aqueous and hydromethanolic extracts from the aerial parts of Moroccan CS and CM were investigated. Antioxidant activity has been assessed using 1,1-diphenyl-2-picrylhydrazyl (DPPH) and 2,2′-azinobis(3-ethylbenzothiazoline-6-sulfonic acid) diammonium salt (ABTS) radicals and ferric reducing/antioxidant power (FRAP) methods. The *α*-amylase and *α*-glucosidase inhibitory activity has been assessed using an in vitro model. Moreover, mineral and phenolic contents of CS and CM were analyzed. The extracts of both species exhibited potent antioxidant activity in all used systems and possess strong inhibitory effect towards *α*-glucosidase (IC_50_: 0.95 ± 0.14 to 14.58 ± 1.26 *μ*g/mL) and significant inhibitory potential against *α*-amylase (IC_50_: 217.10 ± 0.15 to 886.10 ± 0.10 *μ*g/mL). Furthermore, the result showed high levels of phenolic content and unexpectedly some higher levels of mineral content in CS. The results suggest that the phenolic rich extracts of CS and CM may have a therapeutic potential against diseases associated with oxidative stress and may be useful in the management of hyperglycemia in diabetic patients.

## 1. Introduction

Plants have been the basis for medical treatments through much of the human history. Nowadays, researchers are increasingly interested in medicinal plants as alternative medicine, due to their good pharmacological properties, fewer side effects, and low cost. The genus* Cistus *L. (Cistaceae) comprises many interesting medicinal plants, distributed primarily in the Mediterranean region. Among them, twelve species are members of Moroccan flora [1].* Cistus* species are frequently used in traditional medicines for the treatment of hyperglycemia and diabetes [2, 3], peptic ulcers, and diarrhea and also as general remedies for several skin diseases and as anti-inflammatory and antispasmodic agents [4]. Furthermore, phytochemical studies on different* Cistus* species have revealed the presence of several phenolic compounds mainly flavonoids and tannins [5–9]. Those compounds are generally involved in many biological activities, essentially in oxidative stress prevention.

Diabetes mellitus is a serious chronic metabolic disorder that causes serious health complications and is a major cause of mortality [10]. Excessive postprandial glucose excursions are a known risk factor for developing diabetes [11]. One interesting approach for limiting the excursion is to inhibit the activity of digestive enzymes of glucose production such as *α*-amylase and *α*-glucosidase [12].

Despite the great scientific interest in* Cistus* genus currently,* C. salviifolius *L. and* C. monspeliensis *L*.,* which are among the most abundant species in Morocco, remain undiscovered and underinvestigated. To the best of our knowledge, there are no previous reports of any in vitro *α*-amylase and *α*-glucosidase inhibitory effects and antioxidant activities of Moroccan* C. salviifolius *L. and* C. monspeliensis *L. Therefore, the objective of this study was to evaluate the in vitro antioxidant *α*-amylase and *α*-glucosidase inhibitory potentials of aqueous and hydromethanolic extracts of both species.

## 2. Materials and Methods

### 2.1. Reagents

*p*-Nitrophenyl-*α*-D-glucopyranoside (*p*NPG), *α*-glucosidase from* Saccharomyces cerevisiae,α*-amylase from* Bacillus licheniformis,* acarbose, Folin-Ciocalteu reagent, rutin, catechin, DPPH, ABTS, 6-hydroxy-2,5,7,8-tetramethylchroman-2-carboxylic acid (Trolox), butylated hydroxytoluene (BHT), and ascorbic acid were purchased from Sigma-Aldrich (France). All other reagents were of analytical grade.

### 2.2. Plant Material

*Cistus salviifolius *L. and* Cistus monspeliensis *L. were collected in April 2015 from Maâmoura Forest, Salé (CS), and Maaziz-Khémisset (CM) in Morocco. Voucher specimens “RAB 1012176” and “RAB 1012177” for CS and CM, respectively, were deposited in the Herbarium of Botany Department of the Scientific Institute of Rabat, Morocco. The aerial parts were cleaned and dried in the shade at room temperature until reaching a constant weight and were then powdered and used for further investigation.

### 2.3. Preparation of Plant Extracts

For hydromethanolic extract preparation (hydromethanolic extract of* Cistus salviifolius* (CSM) and hydromethanolic extract of* Cistus monspeliensis* (CMM)), 50 g of dried sample was extracted with 500 mL of 80% aqueous methanol at room temperature and under mechanical stirring for 24 hours. Aqueous extracts of* Cistus salviifolius* (CSA) and of* Cistus monspeliensis* (CMA) were prepared with the same ratio in boiling water and allowed to cool for one hour. The extracts were then filtered on Whatman paper and the filtrate obtained was evaporated under reduced pressure, using a rotary evaporator.

### 2.4. Mineral Analysis

CS and CM mineral composition (Ca, Cu, Mg, Fe, K, Mg, Na, P, and Zn) was determined using inductively coupled plasma atomic emission spectroscopy (ICP AES, Jobin Yvon Ultima 2) as previously described [13]. Briefly, 150 mg of the aerial parts powder was etched with 2 mL of HNO_3_ acid (70%) mixture in a teflon beaker, before being incinerated at 110°C. Then, 0.5 mL of hydrofluoric acid (HF) was added and the covered beaker was placed on a sand bath. The sample mixture was heated until a clear solution was obtained. After removing the cover, the mixture was evaporated until drying. Finally, 2 mL of HCl acid was added and the residue was extracted by 25 mL of 2.0 M HCl.

### 2.5. Determination of Total Phenolic Content

Total phenolic content of aqueous and hydromethanolic extracts of CS and CM was determined by the method described by Spanos and Wrolstad [14] and modified by Lister and Wilson [15] using Folin-Ciocalteu reagent. The 0.5 mL of sample solution was mixed with 2.5 mL of Folin-Ciocalteu reagent (previously diluted with distilled water 1 : 10 v/v) and 4 mL of sodium carbonate (7.5% w/v). The mixture is then incubated in a water bath at 45°C for 30 min. The absorbance against blank was determined at 765 nm using a UV-Vis spectrophotometer. Gallic acid (0.487–31.25 *μ*g/mL) was used to perform the standard curve. The results were expressed as mg of gallic acid equivalents per gram of extract dry weight (mg GAE/g edw).

### 2.6. Determination of Total Flavonoid Content

The total flavonoid content was determined according to the method described by Dewanto et al. [16]. Briefly, 1 mL of dissolved sample was placed in a 10 mL volumetric flask. Distilled water was added to make the volume reach 7.4 mL and then 0.3 mL of NaNO_2_ (5%) was added. 0.3 mL of AlCl_3_ (10% w/v) was added 5 min later. After 6 min, 2 mL of 1 M NaOH was added and the solution was mixed well and allowed to stand for 30 min. The absorbance was recorded against a blank at 510 nm. Rutin (50–400 *μ*g/mL) was used as a standard for constructing the calibration curve. The flavonoid content was expressed as rutin equivalent per gram of extract dry weight (mg RE/g edw).

### 2.7. Determination of Proanthocyanidin Content

The procedure reported by Julkunen-Tiitto [17] was used to determine the proanthocyanidin content in our extracts. Aliquots of 50 *μ*L of the extract were mixed with 1.5 mL of 4% vanillin solution (prepared with methanol) and then 750 *μ*L of concentrated HCl was added. The well mixed solution was incubated at ambient temperature in the dark for 20 min. (+)-Catechin (50 *μ*g–1000 *μ*g/mL) was used to make the standard curve, and the results were expressed as catechin equivalent per gram of extract dry weight (mg CE/g edw).

### 2.8. Antioxidant Activities

#### 2.8.1. DPPH Radical Scavenging Activity Assay

Radical scavenging activity of the extracts was measured using the stable radical DPPH by Huang et al. [18]. A solution of DPPH (0.2 mM) was prepared, and 0.5 mL of this solution was mixed with 2.5 mL of the extracts (1.33–1.66 *μ*g/mL). The reaction mixture was vortexed thoroughly and left in the dark at room temperature for 30 min. The absorbance of the mixture was measured at 517 nm in a spectrophotometer. Lower absorbance of the reaction mixture indicated higher free radical scavenging activity. The BHT (0.48–4.76 *μ*g/mL) was used as reference compound. The capability to scavenge the DPPH radical was calculated using the following equation:

(1) $$\begin{array}{c} \text{DPPH scavenging effect }\left(\;\%\;\right)=\left(\;\frac{\left(A_{0}-A_{1}\right)}{A_{0}}\;\right)\times 100, \end{array}$$

where *A*_0_ is the absorbance of the control reaction and *A*_1_ is the absorbance of the sample solution or standard, and the experiment was carried out in triplicate. Scavenging activity in this assay was expressed as IC_50_, which represents the concentration of the extract required to inhibit 50% of the free radical scavenging activity.

#### 2.8.2. ABTS Radical Scavenging Assay

The ability of our extracts to scavenge the ABTS radical was determined according to the previously described method [19]. A solution of ABTS radical cation (ABTS^*∙*+^) was prepared by the reaction between 10 mL of 2 mM ABTS in H_2_O and 100 *μ*L of 70 mM potassium persulphate at room temperature in the dark for 24 h. The ABTS^*∙*+^ solution was then diluted with methanol to obtain absorbance of 0.70 at 734 nm. Samples were prepared in triplicate by diluting 200 *μ*L of extracts in 2 mL of the ABTS^*∙*+^ solution diluted with methanol and allowed to react for 1 min. The absorbance was recorded on a spectrophotometer at 734 nm. The antioxidant activities of samples were expressed as TEAC values, defined as the concentration of standard Trolox with the same antioxidant capacity of the extract under investigation. The results were represented as Trolox equivalent per gram of extract dry weight (mg TE/edw).

#### 2.8.3. Ferric Reducing/Antioxidant Power (FRAP) Assay

The reducing power was assayed as previously described [20] with some modifications. In brief, the extract (1 mL) was mixed with 2.5 mL of phosphate buffer (0.2 M, pH 6.6) and 2.5 mL of 1% potassium ferricyanide. The mixture was then incubated at 50°C for 20 min. Then, 2.5 mL of trichloroacetic acid (10%) was added to the mixture, which was then centrifuged at 3000 rpm for 10 min. Finally, 2.5 mL of the supernatant was mixed with 2.5 mL of distilled water and 0.5 mL FeCl_3_ solution (0.1%, w/v). The absorbance was measured at 700 nm. Increased absorbance values indicate a higher reducing power. The results were expressed as ascorbic acid equivalent per gram of extract dry weight (mg AAE/g edw).

### 2.9. *α*-Amylase Inhibitory Assay

The *α*-amylase inhibitory potentials were investigated by reacting different concentrations of the extracts with *α*-amylase enzyme and starch solution, according to the previously described method [21] with slight modifications. A mixture of 250 *μ*L of samples and 250 *μ*L of 0.02 M sodium phosphate buffer (pH = 6.9) containing the enzyme *α*-amylase (240 U/mL) was incubated at 37°C for 20 min. Then, 250 *μ*L of 1% starch solution in 0.02 M sodium phosphate buffer (pH = 6.9) was added to the reacting mixture. Therefore, the reaction mixture was incubated at 37°C for 15 min. Thereafter, 1 mL of dinitrosalicylic acid (DNS) was added, and the reaction mixture was incubated in a boiling water bath for 10 min. Then, the reaction mixture was diluted by adding 2 mL of distilled water, and absorbance was measured at 540 nm in the spectrophotometer. Acarbose was used as positive control.

The results were expressed as percentage inhibition and calculated using the following formula:

(2) $$\begin{array}{c} \text{inhibition }\left(\;\%\;\right)=\left[\;\frac{\left(A_{\text{c}}-A_{\text{cb}}\right)-\left(A_{\text{s}}-A_{\text{sb}}\right)}{\left(A_{\text{c}}-A_{\text{cb}}\right)}\;\right]\times 100, \end{array}$$

where *A*_c_ refers to the absorbance of control (enzyme and buffer); *A*_cb_ refers to the absorbance of control blank (buffer without enzyme); *A*_s_ refers to the absorbance of sample (enzyme and inhibitor); and *A*_sb_ is the absorbance of sample blank (inhibitor without enzyme). Moreover, IC_50_ values (concentration of inhibitor required to inhibit 50% of enzyme activity) were determined.

### 2.10. *α*-Glucosidase Inhibitory Assay

The *α*-glucosidase inhibitory activity of the extracts was determined using the substrate* p*NPG according to the method described by Kee et al. [22], with some modification. Briefly, a mixture of 150 *μ*L of the samples and 100 *μ*L of 0.1 M sodium phosphate buffer (pH = 6.7) containing the enzyme *α*-glucosidase solution (0.1 U/mL) was incubated at 37°C for 10 min. After preincubation, 200 *μ*L of 1 mM* p*NPG solution in 0.1 M sodium phosphate buffer (pH = 6.7) was added. The reaction mixtures were incubated at 37°C for 30 min. After incubation, 1 mL of 0.1 M of Na_2_CO_3_ was added and the absorbance was recorded at 405 nm using the spectrophotometer.

The *α*-glucosidase inhibitory activity was expressed as percentage inhibition, and the IC_50_ values were determined. Acarbose was used as positive control.

### 2.11. Statistical Analysis

Data were indicated as the mean ± standard error. The data were analyzed by one-way analysis of variance (one-way ANOVA). Post hoc procedure was used for significance of difference. A difference in the mean values of *p* < 0.05 was considered to be statistically significant. Analysis was performed with GraphPad Prism 6.

## 3. Results and Discussion

### 3.1. Mineral Content

Mineral contents in aerial parts of CS and CM, expressed in mg/kg, are shown in Table 1. Five macroelements (calcium (Ca), potassium (K), magnesium (Mg), sodium (Na), and phosphorus (P)) and tree microelements (copper (Cu), iron (Fe), and zinc (Zn)) were analyzed. CS has significantly (*p* < 0.05) higher contents of Ca (3684.32 ± 30.21 mg/kg), Mg (785.27 ± 14.44 mg/kg), K (287.84 ± 3.13 mg/kg), P (275.26 ± 5.64 mg/kg), Na (175.97 ± 1.93 mg/kg), and Cu (84.00 ± 9.95 mg/kg) in comparison to CM, while CM has significantly higher amounts of Fe (29.16 ± 1.17 mg/kg); there is no statistically significant difference between CS and CM in Zn content. The differences in mineral contents are probably linked to genetic profile and partially to environmental conditions. The present study revealed that CS and CM are a good source of Ca, Mg, P, Na, and K, which are very important in human nutrition. To our knowledge, there is no previous report on mineral content of CS and CM. Nevertheless, it has been reported that* Cistus *species (*Cistus ladanifer *L. and* Cistus libanotis *L.) from Morocco showed high levels of some mineral elements [23].

### 3.2. Total Phenolic, Flavonoid, and Proanthocyanidin Contents

Total phenolic, flavonoid, and proanthocyanidin contents are presented in Table 2. The phenolic contents in aqueous and hydromethanolic extracts of CS were found to be 408.43 ± 1.09 mg GAE/g edw and 336.51 ± 1.22 mg GAE/g edw, respectively, which are significantly (*p* < 0.05) greater than the aqueous and hydromethanolic extracts of CM (261.76 ± 1.93 mg GAE/g edw and 282.53 ± 0.58 mg GAE/g edw), respectively. Results of flavonoid and proanthocyanidin contents show that hydromethanolic extraction resulted in significantly (*p* < 0.05) higher values of those compounds in both species. The amounts of flavonoid and proanthocyanidin in hydromethanolic extracts of CS and CM, respectively, were 188.66 ± 2.90 mg RE/g edw, 168.30 ± 3.03 mg CE/edw, 154.00 ± 2.30 mg RE/g edw, and 188.99 ± 7.13 mg CE/g edw, while in aqueous extracts they were 140.00 ± 1.15 mg RE/g edw, 154.15 ± 3.31 mg CE/g edw, 78.00 ± 1.15 mg RE/g edw, and 151.42 ± 0.94 mg CE/g edw, for CSA and CMA, respectively. The interesting phenolic contents of both plants indicate an important health promoting activity. Those compounds are secondary plant metabolites involved in the normal growth and development and act as defense mechanisms of plants against pathogenic and parasite infection and free radicals generation. Phenolic compounds have been reported to have multiple biological effects and are considered as a major group of chemicals that contribute to the antioxidant potential of plant extracts.

### 3.3. Antioxidant Activity

Free radicals and other reactive oxygen species are constantly formed in the human body. They have been implicated in various pathological conditions involving cardiovascular disease, cancer, neurological disorders, diabetes, ischemia/reperfusion, and other diseases and ageing [24]. Despite the effectiveness of synthetic antioxidants, their use is associated with serious adverse effects on health [25, 26]; therefore, the use of medicinal plant extracts as a potential source of antioxidant with limited or no side effects is an important alternative way to operate.

To investigate the antioxidant activity of our plant extracts, we evaluated their abilities to scavenge the stable free radical DPPH and the cation ABTS and their ferric reducing antioxidant power (FRAP). The DPPH is one of the stable and commercially available organic nitrogen radicals and has a strong absorption band at 517 nm [27]. This free radical converges from purple to yellow by accepting a lone pair of electrons or hydrogen radical. The scavenging effects of aqueous and hydromethanolic extracts on DPPH radicals were expressed by IC_50_ and illustrated in Table 2. The result showed that all extracts are likely to have excellent scavenging effects of DPPH free radicals. The* Cistus* extracts activity showed almost similar IC_50_ to BHT (3.28 ± 0.59 *μ*g/mL) which is a well known antioxidant. The hydromethanolic extract CSM (IC_50_ = 3.30 ± 0.25 *μ*g/mL) exhibits significantly (*p* < 0.05) higher DPPH radical scavenging activity.

In the ABTS assay, the antioxidant reduces the cation ABTS^•+^ to ABTS resulting in its decolorization. The ability of the extracts to scavenge ABTS cation was expressed in Table 2. All tested extracts had a strong capacity to quench ABTS^•+^; the extract CSM still had significantly (*p* < 0.05) the highest activity (259.50 ± 0.43 mg TE/g edw).

Moreover, in the FRAP method, the yellow color of the test solution changes to various shades of green and blue, depending on the reducing power of each sample. The presence of reducers causes the conversion of the Fe^3+^/ferricyanide complex used in this method to the ferrous form [28]. Reducing power of CS and CM extracts was interestingly considerable compared to ascorbic acid. The reducing power of hydromethanolic and aqueous extracts is represented in Table 2. In this assay, the highest activities were noted for CS extracts, and the aqueous extract of CSA (650.26 ± 1.76 mg AAE/g edw) had the highest activity followed by hydromethanolic extracts CSM (626.00 ± 0.46 mg AAE/g edw), CMM (610.26 ± 2.26 mg AAE/g edw), and CMA (524.13 ± 1.18 mg AAE/g edw), respectively. The differences between extracts are statistically significant (*p* < 0.05).

Our results showed that the tested plant extracts possessed strong antioxidant activities. A study of correlation between total polyphenolic content and DPPH, ABTS radicals scavenging capacity, and ferric reducing ability shows *r*^2^ values of 0.52, 0.55, and 0.82, respectively. The presence of a high concentration of phenolic compounds in plants extracts may not always translate a high antioxidant capacity; this can be explained by several factors, including the presence of different active compounds that can modify the antioxidant capacity, the synergistic effects of different compounds, and also the fact that antioxidant efficiency of the polyphenols seemed to depend on the position and extent of hydroxylation and conjugation [29].

### 3.4. *α*-Amylase and *α*-Glucosidase Inhibitory Activities

Salivary and pancreatic *α*-amylase catalyzes the hydrolysis of *α*-1,4-glucosidic linkages of polysaccharide such as starch and glycogen. Subsequently, the *α*-glucosidase located in the brush border surface membrane of intestinal cells hydrolyzes the resulting oligosaccharides into glucose, which is then transported into the blood. The inhibition of *α*-amylase and *α*-glucosidase activity in the digestive tract of humans retards absorption of glucose and therefore can be an important strategy in the management of postprandial blood glucose level in diabetic patients [30, 31].

**Figure 1:**
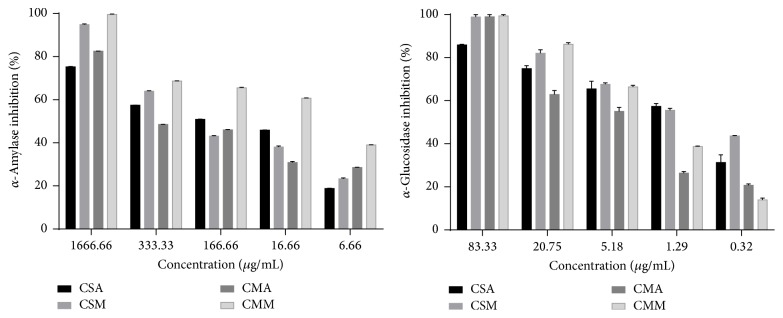
Percentage of *α*-amylase and *α*-glucosidase inhibition versus different concentrations of* Cistus* extracts. CSA: aqueous extract of* Cistus salviifolius*; CSM: methanolic extract of* Cistus salviifolius*; CMA: aqueous extract of* Cistus monspeliensis*; CMM: methanolic extract of* Cistus monspeliensis*.

The *α*-amylase inhibitory property of the aqueous and hydromethanolic extracts of CS and CM is presented in Figure 1. The result revealed that the tested extracts inhibited *α*-amylase activity concentration-dependently (6.66–1666.66 *μ*g/mL). Furthermore, all extracts showed significantly (*p* < 0.05) better activity than the reference compound acarbose (IC_50_ = 311.20 ± 1.38 *μ*g/mL) (Table 3). The extract CSA has the highest inhibitory activity against *α*-amylase with IC_50_ value of 217.10 ± 0.15 *μ*g/mL.

Likewise, the extracts have shown promising and concentration-dependent (0.32–83.33 *μ*g/mL) inhibitory effect on *α*-glucosidase enzyme (Figure 1).

Interestingly, the IC_50_ values 0.95 ± 0.14 *μ*g/mL, 8.47 ± 0.58 *μ*g/mL, 14.58 ± 1.26 *μ*g/mL, and 2.67 ± 0.50 *μ*g/mL for CSA, CSM, CMA, and CMM, respectively, indicate that all tested extracts were significantly (*p* < 0.05) stronger inhibitors of *α*-glucosidase than the reference compound acarbose (IC_50_= 18.01 ± 2.00 *μ*g/mL) (Table 3).

The significant inhibitory effects of CS and CM extracts against the enzymes *α*-amylase and *α*-glucosidase demonstrate their potential abilities to reduce the postprandial increase of blood glucose levels in diabetic patients and their capacities to prevent type 2 diabetes.

Our finding is in accordance with earlier reports that showed that some medicinal plant extracts have more potent *α*-glucosidase inhibitory activities than powerful synthetics inhibitors such as acarbose [32–34]. Also, the results are in line with a study performed on another species of* Cistus* genus (*Cistus laurifolius *L.) that showed that ethanolic extract of this plant is a potent inhibitor of *α*-glucosidase (IC_50_ = 6.3 *μ*g/mL) and has a remarkable and dose-dependent inhibitory effect on *α*-amylase and also improves hyperglycemia in type 2 diabetic rats [35].

Phytochemical studies on CS and CM demonstrated their abilities to produce a high amount of phenolic compounds and several flavonoids, including quercetin, myricetin, and kaempferol. Their glycosylated derivatives were isolated from the aerial parts of* C. salviifolius* plants [9]. Also, several catechin, epicatechin, gallocatechin, and epigallocatechin and their derivatives were isolated from the air-dried CS herb [5]. Likewise, catechin-related compounds [6, 8], apigenin diglucoside, myricetin, gallic and phenolic acids [36], and numerous diterpenes of labdane and clerodane types [37] were identified in CM.

A study of the correlation between total phenolic content and inhibition of *α*-amylase and *α*-glucosidase activities by the investigated extracts had *r*^2^ values of 0.98 and 0.64, respectively, indicating that the phenolic compounds present in the extracts are potentially responsible for the inhibition of *α*-amylase and *α*-glucosidase activity. This is not an unexpected finding, since the phenolic compounds are known by their ability to inhibit the activities of carbohydrate-hydrolyzing enzymes due to their ability to bind with proteins [38]. Also, the flavonoids, different types of catechin, and terpenoids identified in these plants have been known to possess high inhibitory potential towards *α*-amylase and *α*-glucosidase enzymes activity [39, 40].* Cistus* species are used in many traditional medicines to treat several pathological conditions, including hyperglycemia and diabetes [2, 3]. In general, herbal therapy is based on therapeutic action of complex mixtures of different compounds that often act in a synergistic mode to exert their full beneficial effects. This suggests that the biologically active compounds present in the investigated extracts may be acting in a synergistic therapeutic fashion to exert their carbohydrate-hydrolyzing enzymes inhibition activities and antioxidant effects.

Currently, the continuous use of synthetics *α*-amylase and *α*-glucosidase inhibitors such as acarbose is often associated with undesirable side effects such as abdominal distention, diarrhea, and flatulence [41, 42]. Additionally, only few of them are commercially available and their synthesis involves a tedious multistep procedure. Therefore, the CS and CM extracts with strong inhibitory activity against *α*-glucosidase and significant inhibitory activity on *α*-amylase enzyme may be effective therapeutic agents for the control of hyperglycemia and offer an attractive target to discover new agents for treatment of diabetes mellitus with minimal side effects.

## 4. Conclusion

The aqueous and hydromethanolic extracts of the aerial parts of* Cistus salviifolius *L. and* Cistus monspeliensis *L. presented high antioxidant effects against the DPPH and ABTS radicals and a strong ferric reducing power compared to the synthetic antioxidants analyzed. Those results suggest that the CS and CM extracts may have therapeutic potential against diseases associated with oxidative stress. Likewise, the interesting potential of investigated extracts to inhibit *α*-glucosidase enzyme and their significant inhibition of *α*-amylase indicate that they may be effective therapeutic agents for controlling hyperglycemia and bring about some preliminary proofs for their antidiabetic effects. Additionally, it appears that both plants are a promising source of bioactive compounds, since those activities seem to be linked to the phenolic content, and it is noteworthy that the mineral contents of both plants may contribute significantly to their health promoting properties.

## Figures and Tables

**Table 1:** Mineral composition of *Cistus *expressed as mg/kg.

Minerals	*Cistus salviifolius*	*Cistus monspeliensis*
Ca	3684.32 ± 30.21^b^	475.32 ± 5.13^a^
Cu	84.00 ± 9.95^b^	17.68 ± 0.19^a^
Fe	2.95 ± 0.17^a^	29.16 ± 1.17^b^
K	287.84 ± 3.13^b^	27.87 ± 0.90^a^
Mg	785.27 ± 14.44^b^	95.78 ± 0.93^a^
Na	175.97 ± 1.93^b^	25.97 ± 0.27^a^
P	275.26 ± 5.64^b^	204.75 ± 4.13^a^
Zn	5.90 ± 0.30^a^	17.48 ± 0.35^a^

Data are reported as mean (*n* = 3) ± standard error.

Values in the same row not sharing a common letter (a and b) differ significantly at *p* < 0.05.

**Table 2:** Total phenolic, flavonoid, and proanthocyanidin contents and antioxidant activities of* Cistus *extracts.

	Phenolic content^(1)^	Flavonoid content^(2)^	Proanthocyanidin content^(3)^	ABTS^(4)^	FRAP^(5)^	DPPH^(6)^
CSA	408.43 ± 1.09^d^	140.00 ± 1.15^b^	154.15 ± 3.31^a^	256.82 ± 0.43^b^	650.26 ± 1.76^d^	4.10 ± 0.85^b^
CSM	336.51 ± 1.22^c^	188.66 ± 2.90^d^	168.30 ± 3.03^b^	259.50 ± 0.43^c^	626.00 ± 0.46^c^	3.30 ± 0.25^a^
CMA	261.76 ± 1.93^a^	78.00 ± 1.15^a^	151.42 ± 0.94^a^	253.83 ± 0.72^a^	524.13 ± 1.18^a^	5.11 ± 0.17^b^
CMM	282.53 ± 0.58^b^	154.00 ± 2.30^c^	188.99 ± 7.13^c^	255.82 ± 0.88^b^	610.26 ± 2.26^b^	4.18 ± 0.26^b^

The results are expressed as ^(1)^mg of gallic acid equivalent, ^(2)^mg of rutin equivalent, ^(3)^mg of catechin equivalent, ^(4)^mg of Trolox equivalent, ^(5)^mg of ascorbic acid equivalent per gram of extracts dry weight, and ^(6)^IC_50_ (*µ*g/mL).

The values are the mean of three determinations ± standard error.

Values in the same column not sharing a common letter (a to d) differ significantly at *p* < 0.05.

CSA: aqueous extract of *Cistus salviifolius*; CSM: methanolic extract of *Cistus salviifolius*; CMA: aqueous extract of *Cistus monspeliensis*; CMM: methanolic extract of *Cistus monspeliensis*.

**Table 3:** IC_50_ values of CS and CM extracts on *α*-amylase and *α*-glucosidase inhibition.

	IC_50_ (*µ*g/mL)	
	*α*-Amylase	*α*-Glucosidase
Acarbose	311.20 ± 1.38^b^	18.01 ± 2.00^d^
CSA	217.10 ± 0.15^a^	0.95 ± 0.14^a^
CSM	597.10 ± 0.26^c^	8.47 ± 0.58^b^
CMA	886.10 ± 0.10^e^	14.58 ± 1.26^c^
CMM	706.50 ± 0.17^d^	2.67 ± 0.50^a^

The values are the mean of three determinations ± standard error.

Values in the same column not sharing a common letter (a to e) differ significantly at *p* < 0.05.

CSA: aqueous extract of *Cistus salviifolius*; CSM: methanolic extract of *Cistus salviifolius;* CMA: aqueous extract of *Cistus monspeliensis*; CMM: methanolic extract of *Cistus monspeliensis*.

## References

[1] El Alaoui-Faris F. E., Mrabet N., Tahiri H. (2009). Nombre chromosomique et caryotype de *Cistus ladanifer* subsp. Africanus dansereau (*Cistaceae*). *Lagascalia*.

[2] Bnouham M., Mekhfi H., Legssyer A., Ziyyat A. (2002). Ethnopharmacology Forum Medicinal plants used in the treatment of diabetes in Morocco. *International Journal of Diabetes & Metabolism*.

[3] Polat R., Satil F. (2012). An ethnobotanical survey of medicinal plants in Edremit Gulf (Balikesir—Turkey). *Journal of Ethnopharmacology*.

[4] Attaguile G., Perticone G., Mania G., Savoca F., Pennisi G., Salomone S. (2004). *Cistus incanus* and *Cistus monspeliensis* inhibit the contractile response in isolated rat smooth muscle. *Journal of Ethnopharmacology*.

[5] Danne A., Petereit F., Nahrstedt A. (1994). Flavan-3-ols, prodelphinidins and further polyphenols from *Cistus salvifolius*. *Phytochemistry*.

[6] Pomponio R., Gotti R., Santagati N. A., Cavrini V. (2003). Analysis of catechins in extracts of *Cistus* species by microemulsion electrokinetic chromatography. *Journal of Chromatography A*.

[7] Saracini E., Tattini M., Traversi M. L., Vincieri F. F., Pinelli P. (2005). Simultaneous LC-DAD and LC-MS determination of ellagitannins, flavonoid glycosides, and acyl-glycosyl flavonoids in *Cistus salvifolius* L. leaves. *Chromatographia*.

[8] Santagati N. A., Salerno L., Attaguile G., Savoca F., Ronsisvalle G. (2008). Simultaneous determination of catechins, rutin, and gallic acid in *Cistus* species extracts by HPLC with diode array detection. *Journal of Chromatographic Science*.

[9] Gürbüz P., Demirezer L. Ö., Güvenalp Z., Kuruüzüm-Uz A., Kazaz C. (2015). Isolation and structure elucidation of uncommon secondary metabolites from *Cistus salviifolius* L.. *Records of Natural Products*.

[10] Bailes B. K. (2002). Diabetes mellitus and its chronic complications. *AORN Journal*.

[11] Williamson G. (2013). Possible effects of dietary polyphenols on sugar absorption and digestion. *Molecular Nutrition and Food Research*.

[12] Loizzo M. R., Marrelli M., Pugliese A. (2016). *Crocus cancellatus* subsp. Damascenus stigmas: chemical profile and inhibition of *α*-amylase and *α*-glucosidase and lipase, key enzymes related to type 2 diabetes and obesity. *Journal of Enzyme Inhibition and Medicinal Chemistry*.

[13] Marmouzi I., El Madani N., Charrouf Z., Cherrah Y., Faouzi M. E. A. (2015). Proximate analysis, fatty acids and mineral composition of processed Moroccan *Chenopodium quinoa* Willd. and antioxidant properties according to the polarity. *Phytothérapie*.

[14] Spanos G. A., Wrolstad R. E. (1990). Influence of processing and storage on the phenolic composition of thompson seedless grape juice. *Journal of Agricultural and Food Chemistry*.

[15] Lister E., Wilson P. (2001). *Measurement of Total Phenolics and ABTS Assay for Antioxidant Activity*.

[16] Dewanto V., Wu X., Liu R. H. (2002). Processed sweet corn has higher antioxidant activity. *Journal of Agricultural and Food Chemistry*.

[17] Julkunen-Tiitto R. (1985). Phenolic constituents in the leaves of northern willows: methods for the analysis of certain phenolics. *Journal of Agricultural and Food Chemistry*.

[18] Huang B., Ke H., He J., Ban X., Zeng H., Wang Y. (2011). Extracts of *Halenia elliptica* exhibit antioxidant properties in vitro and in vivo. *Food and Chemical Toxicology*.

[19] Tuberoso C. I. G., Boban M., Bifulco E., Budimir D., Pirisi F. M. (2013). Antioxidant capacity and vasodilatory properties of Mediterranean food: the case of Cannonau wine, myrtle berries liqueur and strawberry-tree honey. *Food Chemistry*.

[20] Oyaizu M. (1986). Studies on products of browning reaction. Antioxidative activities of products of browning reaction prepared from glucosamine. *The Japanese Journal of Nutrition and Dietetics*.

[21] Hashim A., Khan M. S., Khan M. S., Baig M. H., Ahmad S. (2013). Antioxidant and *α*-amylase inhibitory property of *Phyllanthus virgatus* L.: an *in vitro* and molecular interaction study. *BioMed Research International*.

[22] Kee K. T., Koh M., Oong L. X., Ng K. (2013). Screening culinary herbs for antioxidant and *α*-glucosidase inhibitory activities. *International Journal of Food Science & Technology*.

[23] Zidane H., Aouniti F., Tahani A., Fauconnier M. L., Elbachiri A. (2014). Screening of mineral elements in *Cistus ladanifer* and *Cistus libanotis* essential oils and their leaves. *Oriental Journal of Chemistry*.

[24] Valko M., Leibfritz D., Moncol J., Cronin M. T. D., Mazur M., Telser J. (2007). Free radicals and antioxidants in normal physiological functions and human disease. *International Journal of Biochemistry and Cell Biology*.

[25] Williams G. M. (1994). Interventive prophylaxis of liver cancer. *European Journal of Cancer Prevention*.

[26] Shahidi F., Zhong Y. (2010). Novel antioxidants in food quality preservation and health promotion. *European Journal of Lipid Science and Technology*.

[27] Blois M. S. (1958). Antioxidant determinations by the use of a stable free radical. *Nature*.

[28] Gülçin I., Oktay M., Kireçci E., Küfrevioğlu Ö. I. (2003). Screening of antioxidant and antimicrobial activities of anise (*Pimpinella anisum* L.) seed extracts. *Food Chemistry*.

[29] Pietta P.-G. (2000). Flavonoids as antioxidants. *Journal of Natural Products*.

[30] Bhandari M. R., Jong-Anurakkun N., Hong G., Kawabata J. (2008). *α*-Glucosidase and *α*-amylase inhibitory activities of Nepalese medicinal herb Pakhanbhed (*Bergenia ciliata*, Haw.). *Food Chemistry*.

[31] Malapermal V., Botha I., Krishna S. B. N., Mbatha J. N. (2015). Enhancing antidiabetic and antimicrobial performance of *Ocimum basilicum*, and *Ocimum sanctum* (L.) using silver nanoparticles. *Saudi Journal of Biological Sciences*.

[32] Zhang H., Wang G., Beta T., Dong J. (2015). Inhibitory properties of aqueous ethanol extracts of propolis on alpha-glucosidase. *Evidence-Based Complementary and Alternative Medicine*.

[33] Figueiredo-González M., Grosso C., Valentão P., Andrade P. B. (2016). *α*-Glucosidase and *α*-amylase inhibitors from *Myrcia* spp.: a stronger alternative to acarbose?. *Journal of Pharmaceutical and Biomedical Analysis*.

[34] Ortíz-Martinez D. M., Rivas-Morales C., de la Garza-Ramos M. A., Verde-Star M. J., Nuñez-Gonzalez M. A., Leos-Rivas C. (2016). *Miconia* sp. increases mRNA levels of PPAR gamma and inhibits alpha amylase and alpha glucosidase. *Evidence-Based Complementary and Alternative Medicine*.

[35] Orhan N., Aslan M., Sukuroglu M., Deliorman Orhan D. (2013). In vivo and in vitro antidiabetic effect of *Cistus laurifolius* L. and detection of major phenolic compounds by UPLC-TOF-MS analysis. *Journal of Ethnopharmacology*.

[36] Barrajõn-Catalán E., Fernández-Arroyo S., Roldán C. (2011). A systematic study of the polyphenolic composition of aqueous extracts deriving from several Cistus genus species: evolutionary relationship. *Phytochemical Analysis*.

[37] Fokialakis N., Kalpoutzakis E., Tekwani B. L., Skaltsounis A. L., Duke S. O. (2006). Antileishmanial activity of natural diterpenes from Cistus sp. and semisynthetic derivatives thereof. *Biological and Pharmaceutical Bulletin*.

[38] Shobana S., Sreerama Y. N., Malleshi N. G. (2009). Composition and enzyme inhibitory properties of finger millet (*Eleusine coracana* L.) seed coat phenolics: mode of inhibition of *α*-glucosidase and pancreatic amylase. *Food Chemistry*.

[39] Yin Z., Zhang W., Feng F., Zhang Y., Kang W. (2014). *α*-Glucosidase inhibitors isolated from medicinal plants. *Food Science and Human Wellness*.

[40] Tundis R., Loizzo M. R., Menichini F. (2010). Natural products as *α*-amylase and *α*-glucosidase inhibitors and their hypoglycaemic potential in the treatment of diabetes: an update. *Mini-Reviews in Medicinal Chemistry*.

[41] Hollander P. (1992). Safety profile of acarbose, an *α*-glucosidase inhibitor. *Drugs*.

[42] Toeller M. (1994). *α*-glucosidase inhibitors in diabetes: Efficacy in NIDDM subjects. *European Journal of Clinical Investigation, Supplement*.

